# The Dynamics of Decision Making in Risky Choice: An Eye-Tracking Analysis

**DOI:** 10.3389/fpsyg.2012.00335

**Published:** 2012-10-01

**Authors:** Susann Fiedler, Andreas Glöckner

**Affiliations:** ^1^Max Planck Institute for Research on Collective GoodsBonn, Germany

**Keywords:** risky choices, decision field theory, heuristics, parallel constraint satisfaction, eye tracking, arousal, gaze-cascade effect

## Abstract

In the last years, research on risky choice has moved beyond analyzing choices only. Models have been suggested that aim to describe the underlying cognitive processes and some studies have tested process predictions of these models. Prominent approaches are evidence accumulation models such as decision field theory (DFT), simple serial heuristic models such as the adaptive toolbox, and connectionist approaches such as the parallel constraint satisfaction (PCS) model. In two studies involving measures of attention and pupil dilation, we investigate hypotheses derived from these models in choices between two gambles with two outcomes each. We show that attention to an outcome of a gamble increases with its probability and its value and that attention shifts toward the subsequently favored gamble after about two thirds of the decision process, indicating a gaze-cascade effect. Information search occurs mostly within-gambles, and the direction of search does not change over the course of decision making. Pupil dilation, which reflects both cognitive effort and arousal, increases during the decision process and increases with mean expected value. Overall, the results support aspects of automatic integration models for risky choice such as DFT and PCS, but in their current specification none of them can account for the full pattern of results.

## Introduction

In many every day decisions individuals choose between options with different outcomes, each of which realizes with a certain probability. Conceptually, such risky choices can be reduced to decisions between gambles with monetary outcomes. Research in risky choice has a long tradition, and several models have been developed to account for the wealth of identified choice anomalies and data observed on risky choices in the lab and the field. Many of these models are extensions and refinements of the expected utility (EU) Model (von Neumann and Morgenstern, 1944; Edwards, 1954; Savage, 1954) assuming that some kind of integration of payoff and probability of outcomes drives individuals’ choice. Cumulative prospect theory (CPT, Tversky and Kahneman, 1992), and the transfer-of-attention exchange model (Birnbaum, 2008a) are two of the most prominent models of this class. One important limitation of these models, however, is that they predict choices only and remain largely silent concerning the processes underlying choice (Luce and Raiffa, 1957; Luce, 2000).

### Process models for risky choice

The importance of investigating the underlying processes in more depth and of developing process models, however, has been highlighted repeatedly (e.g., Payne et al., 1988; Johnson et al., 2008; Franco-Watkins and Johnson, 2011; Schulte-Mecklenbeck et al., 2011). Two important classes of existing process models are simplifying heuristic models and automatic integration models. Simplifying heuristic models assume that individuals try to avoid integrating all pieces of information and apply short-cuts instead. According to a satisficing heuristics, for example, one could choose the first gamble that meets the criteria for adequacy on all outcomes (Simon, 1955, 1956). Alternatively, one might apply a minimax heuristic or a maximax heuristic, by choosing the gamble which is better then all alternatives concerning its worst outcome, or better in the best outcome, respectively. Persons might also apply a strategy that tests a sequence of such “reasons” as assumed by the priority heuristic (PH; Brandstätter et al., 2006). PH assumes that individuals compare reasons in the order: minimum outcomes (higher is better), probability of the minimum outcome (lower is better), and maximum outcome (higher is better). Participants consider these reasons in turn, make their decision as soon as a reason discriminates between the gambles, and decide in line with this reason.

Automatic integration models, in contrast, assume that individuals rely on powerful cognitive processes that allow integration of much information very quickly and efficiently (Schneider and Shiffrin, 1977; Shiffrin and Schneider, 1977). Examples for automatic integration models in the domain of risky choice are evidence accumulation models such as decision field theory (DFT, Busemeyer and Townsend, 1993; Johnson and Busemeyer, 2005), and the leaky competing accumulator model (Usher and McClelland, 2001, 2004); as well as interactive activation models such as the parallel constraint satisfaction (PCS) model for risky choice (Glöckner and Herbold, 2011; see also Glöckner and Betsch, 2008b; Betsch and Glöckner, 2010). Further automaticity-based evidence accumulation models that focus on decision making under certainty but could eventually also be applied to risky decisions are multiattributive DFT (e.g., Diederich, 1997, 2003), and the attention drift-diffusion model (e.g., Krajbich et al., 2010, 2012; Milosavljevic et al., 2010; Krajbich and Rangel, 2011).

### Model comparisons and tests of process models

A recent comprehensive model comparison indicates that none of the available simple heuristics (not even when assuming their adaptive usage) can predict risky choice in decisions between two-outcome gambles nearly as well as CPT (Glöckner and Pachur, 2012)^1^. Despite of some initial support for heuristics and particularly for the PH (Brandstätter et al., 2006), most later studies have challenged predictions derived from PH and similar semi-lexicographic heuristics with regard to choices (Birnbaum, 2008b; Birnbaum and LaCroix, 2008; Hilbig, 2008), decision times (Glöckner and Betsch, 2008a; Ayal and Hochman, 2009; but see Brandstätter et al., 2006), and information search (Glöckner and Betsch, 2008a; Johnson et al., 2008).

One study also used eye-tracking to directly compare predictions of PH and automatic integration models with regard to the process measures (Glöckner and Herbold, 2011). The results concerning information search, response time, and choice speak against the usage of PH as well as similar heuristics. The findings, however, also rule out that individuals use simple serial implementation of EU models assuming a stepwise calculation of weighted sums, which is one possible process implementation of EU suggested in previous research (Payne et al., 1988). Instead, results support automatic integration models such as DFT and PCS in that mainly short fixations were found, which indicate lower-level automatic processing instead of deliberate calculation (Horstmann et al., 2009). Furthermore, the decision time and number of fixations increased for decisions in which differences between gambles were small. In line with PCS predictions, there was an increase in the number of fixations to the favored gamble and particularly to the most attractive outcome of this gamble (defined by a high product of outcome and probability). Furthermore, and in line with previous studies using mouselab (Glöckner and Betsch, 2008a; Johnson et al., 2008), information search was conducted mainly within-gambles. Most simple heuristics assume attribute-wise comparisons between gambles and therefore cannot account for the information search behavior.

### Aim of the current studies

The current paper elaborates the eye-tracking approach by Glöckner and Herbold for detailed investigations of the dynamics in risky choice, that is, changes of process variables over the time course of a decision. Furthermore, we investigate changes in pupil size as further dependent measure which indexes both processing load (Beatty, 1982) and arousal (Partala and Surakka, 2003; Bradley et al., 2008) and can be informative for cognitive processes in decision research as well (cf. Franco-Watkins and Johnson, 2011). Finally, we aimed to go beyond Glöckner and Herbold (2011) by using more detailed analyses of the factors influencing attention and by additionally addressing the question whether individuals react rather homogeneously or heterogeneously on these influence factors. By putting the decision process under the microscope, we aim to improve our knowledge concerning the mechanisms underlying risky decision making.

### Previous findings concerning dynamics and arousal in decision making

Some previous studies have investigated the dynamics of decision making using eye-tracking in situations under certainty (i.e., outcomes are certain in contrast to probabilistic). A *gaze-cascade effects*, that is the tendency that over the course of decision making attention shifts to the chosen option, has been repeatedly found in these studies. Gaze-cascade effects, have for example been demonstrated in attractiveness based decisions between faces (Shimojo et al., 2003) and other kinds of visual decision task (e.g., Glaholt and Reingold, 2009a,b). Similarly, a recent eye-tracking investigation of food choices testing predictions of the attention drift-diffusion model showed that the last fixated item was chosen more often than the alternative item (Krajbich et al., 2010). Additionally, it was shown in this study that decision time and the number of fixations decrease with the increase in the difference in valuations of the food items measured in a pre-test.

There is one recent eye-tracking paper comparing risky decisions from description and from experience (Glöckner et al., 2012). The former are decisions between gambles with stated probabilities and outcomes as the ones used in the current study. In decisions from experience, in contrast, no probabilities are provided and outcomes have to be sampled sequentially (e.g., Camilleri and Newell, 2011). In the description condition, arousal measured by pupil dilation and skin conductance response increased with the average expected value (EV) of the two gambles and with decreasing difference in EV between gambles. This effect was not found in the experience condition. Based on this study and other studies showing differences in choice behavior (e.g., Barron and Erev, 2003; Hertwig et al., 2004; Ungemach et al., 2009), we restrict the current investigation to decisions from description only. Finally a recent investigation by Franco-Watkins and Johnson (2011) shows that the pupil dilation increases over the course of the decision making and is influenced by the presentation format (basic eye-tracking vs. decision moving-window). Another eye-tracking study using a somewhat different card gambling task shows that in situations in which persons can form expectations pupil dilation signals surprise and not expected reward or uncertainty *per se* (Preuschoff et al., 2011).

## Process Measures and Theories

In the following process analysis, we investigate decision time, number of fixations, distribution of attention, mean fixation duration, pupil dilation, and direction of information search as dependent measures. First, we investigate these measures in an overall perspective aggregated over time for entire decisions (i.e., similar to most analyses in Glöckner and Herbold, 2011). Second, we look at developments and changes in these variables over the time course of making a decision by splitting up the decision process in several parts (i.e., time bins).

### Dependent measures

Decision time was measured as the time from the gamble onset to individuals choice response. The “number of fixations” refers to the average fixation count in this period (per decision of each person). The dependent variable “distribution of attention” refers to the proportion of fixations to specific parts of the screen containing probability or outcome information, so-called areas of interest (AOI). AOIs can thereby also be combinations of smaller areas such as all areas that contain pieces of information belonging to the left or the right gamble. The variable “mean fixation duration” refers to the average duration of single fixations in a decision. Stated differently, it refers to how long each fixation was on average. It has been shown that mean fixation durations increase with level of processing in scene perception in driving (Velichkovsky et al., 2001). This finding generalizes to decision making in that deliberate processes of calculating weighted sums go along with long fixations, whereas more intuitive or superficial information processing is accompanied by shorter fixations (Horstmann et al., 2009). The dependent variable “pupil dilation” refers to the difference in pupil size between periods of task processing (i.e., decision trials) and periods of rest (i.e., intertrial intervals/baseline) as a measure of arousal and cognitive load. The measure “direction of search” is defined as proportion of fixation transitions within one gamble as compared to the sum of transitions within and between gambles (details see below).

### Models

In the investigation we particularly consider the models: DFT, PCS, PH, minimax, maximax, LEX, and (for completeness) a deliberate application of EU which we will refer to as weighted additive strategy (WADD). These decision strategies are briefly described in Table 1.

**Table 1:** **Models for risky choice**.

**AUTOMATIC INTEGRATION MODELS**
Decision field theory (DFT): Decision making is based on a dynamic, stochastic process. Each moment in the choice process is akin to mentally sampling one of these outcomes, producing affective reactions to the imagined result which are added up until a threshold for deciding for one or the other gamble is reached. Sampling is assumed to reflect the probabilities present in the stimuli, therefore outcome probabilities dictate where attention shifts, but only the outcome values are used in determining the momentary evaluation
Parallel constraint satisfaction (PCS): Decision making is based on a dynamic process of constructing coherence under parallel consideration of all constraints given by outcome-probability relations in a decision task between gambles. In a process akin to Gestalt-construction in perception, activation of information supporting the favored gamble is automatically increased, while activation of information speaking for the alternative is decreased to form a coherent interpretation. The option with the higher activation feels more attractive and is more likely to be chosen
**SIMPLE HEURISTICS**
Priority heuristic (PH): Decision making is based on simple set of rules: (1) Go through reasons in the order of: minimum gain, probability of minimum gain, and maximum gain. (2) Stop examination if the minimum gains differs by 1/10 (or more) of the maximum gain; otherwise, stop examination if probabilities differ by 1/10 (or more) of the probability scale. (3) Choose the gamble with the more attractive gain (probability)
Minimax: Decision making is based on a simple rule of choosing the gamble with highest minimum outcome
Maximax: Decision making is based on a simple rule of choosing the gamble with the highest outcome
Lexicographic (LEX): Determine the most likely outcome of each gamble and choose the gamble with the better outcome. If both outcomes are equal, determine the second most likely outcome of each gamble, and choose the gamble with the better (second most likely) outcome. Proceed until a decision is reached
**SERIAL EXPECTATION MODEL**
Weighted additive strategy (WADD). Multiple outcomes by their probability and add them up for each gamble. Choose the gamble with the higher sum.

*Heuristics are from Brandstätter et al. (2006) and Payne et al. (1993)*.

## Manipulation and Predictions

Not all models allow for straightforwardly deriving predictions concerning all dependent variables. Nevertheless, to foster improvements and specifications of models based on our data, we tried to derive as many reasonable predictions as possible (for a detailed discussion of specification and model development see Glöckner and Betsch, 2011). In cases in which reasonable predictions could be derived on theoretical grounds, we did so even if the authors of the original models did not explicitly make these predictions (which is of course explicitly acknowledged). Furthermore, we report data relatively broadly, so that the reader has additionally the possibility to check his or her own hypotheses.

### Decision time and information search

There are several possibilities to compare models. In the current paper, we refrain from complex comparative model fitting but use the classic method of hypothesis testing. We thereby investigate predictions concerning general differences in dependent variables as well as predictions concerning the effect of manipulations. Specifically, we manipulate the mean EV of gambles (EVmean) and the difference in EVs between gambles (EVdiff). EVmean is basically a manipulation of the stakes involved in the task. A high EVmean should increase motivation since participants can win more money. EVdiff is a measure how similar the gambles are from a rational perspective, and according to some models it should be related to the difficulty of choice. Choices between gambles with very similar EVs are challenging in terms of finding the option with the higher EV whereas in gamble pairs with a high EVdiff the better alternative is easier recognizable. The process models considered make quite distinct predictions concerning whether or not individuals should be influenced by manipulations of these factors. According to DFT and PCS, decision time and number of fixations should increase with higher EVmean since this corresponds to an higher internal decision/coherence threshold within these models. An increase in decision time and number of fixations would also be predicted for decisions between-gamble pairs with low EVdiff, as compared to gamble pairs with high EVdiff, since according to DFT the drift rate towards the threshold is lower in these cases and according to PCS it is much harder to create a coherent interpretation of the task. According to heuristics and WADD, measures should not be influenced by either manipulation, because strategies apply rather simple decision rules or a standardized weighting operation, which are independent of these factors as long as the number of elementary information processes (EIPs; see Newell and Simon, 1972; Payne et al., 1988) necessary to apply the strategy is not influenced by the manipulation.

### Mean fixation duration and pupil dilation

Increasing EVmean and decreasing (absolute) EVdiff could both potentially lead to qualitative changes in information processing in that more thorough, effortful, and deliberate information processing is used. Hence, mean fixation duration might increase. Similarly, individuals might be more aroused and diligent, which should be reflected in increasing pupil size. DFT and PCS can both predict the effects of EVmean on pupil size. According to DFT, a higher EVmean will induce persons to use higher thresholds that necessitate a higher accumulation of affective responses^2^. According to PCS, arousal is dependent on the general level of conflict (or dissonance) in the network (Betsch and Glöckner, 2010; Glöckner et al., 2012; see also Hochman et al., 2010; Glöckner and Hochman, 2011), which can be measured by the networks Hopfield energy (Hopfield, 1982; Read et al., 1997). The level of remaining conflict mainly depends on two factors: the general activation of the network and the structure of the network defined by its’ constraints (i.e., the structure of inhibitory and excitatory links). Increasing EVmean influences the first factor and leads to higher activation and therefore also to higher arousal. In the model this would be captured by the fact that higher *a priori* valuations of outcomes result in stronger links between the general valuation node and the outcome nodes (see Glöckner and Herbold, 2011, Figure 1). Furthermore, PCS predicts increasing pupil size for decreasing EVdiff, which influences the second factors namely the structure of the network and makes it harder to construct a coherent interpretation (Hochman et al., 2010; Glöckner and Hochman, 2011). Heuristics predictions are less influenced by either manipulation. Individuals applying heuristics should not be aware of EV as they ignore parts of the information which would be needed to calculate it. They could, however, anyway realize that gambles are concerned with higher stakes by seeing higher outcomes and therefore at least react to the manipulation of EVmean (see also text footnote 2, above).

**Figure 1:**
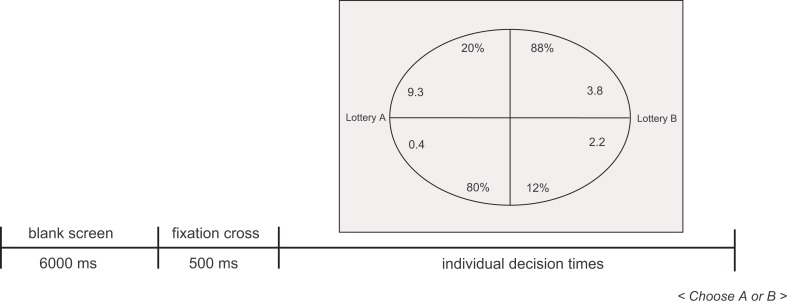
**Decision tasks between gambles with two outcomes each used in studies 1 and 2**. Left outcomes belong to gamble A, right outcomes belong to gamble B.

### Distribution of attention

Most process models allow predictions concerning the distribution of attention over outcomes within a decision. For example, LEX, minimax, and maximax predict that certain outcomes (i.e., low vs. high outcomes) should receive particularly large amounts of attention. For individuals applying WADD, in contrast, all outcomes and probabilities should receive about equal attention. From DFT, even more refined predictions can be derived in that attention can be assumed to be proportional to the probability of an outcome (Busemeyer and Townsend, 1993; Johnson and Busemeyer, 2005)^3^. PCS predicts that attention to outcomes should increase with both their probability and their value. PCS postulates that activation of the aspects supporting the later on favored option increases over time. Therefore, in a dynamic perspective, there should be a shift of attention toward the chosen options (and particularly to the most attractive outcomes of it) in the course of decision making. If one accepts the assumption that internal attention is at least correlated with overt attention in decisions from descriptions (Just and Carpenter, 1976), these predictions can be directly investigated in our fixation data.

### Direction of information search

WADD assumes mainly within-gambles information search. Heuristics, in contrast, mainly assume information search to be between gambles. DFT assumes a stochastic process of information sampling but does not explicitly specify the direction of information search. PCS also does not make clear predictions concerning the direction of information search.

## Materials and Methods

We conducted two eye-tracking studies to investigate the dynamics of risky choice. The first study used variants of the gambles taken from a previous study (Glöckner and Betsch, 2008a)^4^. To rule out the possibility that results might be dependent on the selection of these specific decisions, we conducted a second study using randomly generated gambles. To enhance cross-study comparisons, we will jointly report methods and results for both studies, although the studies were originally conducted in a logically motivated sequence and with a time lag of more than 1 year.

### Participants and design

Twenty-four residents of Bonn, most of them students (mean age: 24.6 years; 47% female), took part in the first study. Three of them had to be excluded from the analysis because their eye-tracking data was not recorded. In the second study, 37 participants took part (mean age: 22.53 years; 65% female). One had to be excluded because of incomplete eye-tracking data. Participants were recruited from the MPI Decision Lab subject pool using the database-system ORSEE (Greiner, 2004). In both studies participants repeatedly made decisions between two gambles with two outcomes each. In the first study, for many of the decisions the compared gambles were almost equal concerning their EV, that is, their EVdifference was close to zero, but the mean EV was manipulated to be high vs. low (details below). In the second study, mean EV and EV differences varied randomly between gambles within a certain pre-specified range.

Both studies lasted about 40 min each including a calibration phase and a short questionnaire about the decision behavior. Choices were incentivized. In addition to a fixed show-up fee of 6 € (approx. USD 7.90), participants earned money by playing one of the chosen lotteries (payoffs ranged from 0 to 49.5 €, *M* = 6.2€ (approx. USD 8.20) in Study 1 and from 0.1 to 49.8 €, *M* = 9.6 € (approx. USD 12.70, in Study 2). No deception was involved, and participants had not experienced deception in previous studies in our lab.

### Material

#### Study 1

Participants completed 50 choices between pairs of gambles. The decision problems used consisted of 40 decision tasks adapted from Glöckner and Betsch (2008a), and 10 decision tasks taken from the standard economic test for risk aversion from Holt and Laury (2002). For the Glöckner-and-Betsch gambles, the mean EV of both gambles in the decision task was manipulated. For half of the decisions, the mean EV for both gambles was high (7.50 € ≤ EVmean ≤ 9.90 €) for the remaining decisions it was low (2.00€ ≤ EVmean ≤ 3.75€). The compared gambles were thereby always similar in EV to each other. In the Holt-and-Laury gambles, in contrast, the absolute difference in EVs between the gambles was varied gradually [0.32 € ≤ EVdiff (abs) ≤ 3.7 €]. Specifically, a safe gamble was compared to a risky gamble with increasing superiority in EV. Mean EV and differences in EVs between the gambles show a slight negative correlation, *r* = −0.17, *p* = 0.23. Decision tasks are listed in Section “Gambles Study 1” in Appendix.

#### Study 2

Decision tasks consisted of the 10 Holt-and-Laury gambles, and 40 decision tasks that were randomly generated. They contained pairs of gambles with varying mean EV (2.04 € ≤ EVmean ≤ 17.6 €) and varying the absolute difference in EV between gambles [0.006 € ≤ EVdiff (abs) ≤ 1.844 €]. Both factors were thereby almost uncorrelated (*r* = −0.04). The full list of decision problems can be found in Section “Gambles Study 2” in Appendix.

### Procedure

Both studies essentially used the same procedure. First, participants were informed about the experimental task, the incentive scheme, and the presentation format of the gambles. They were instructed to make good decisions, but to proceed as fast as possible. Each decision started with a blank screen (6 s), followed by the fixation cross (0.5 s) to direct attention to the center of the screen. Then the two gambles were presented simultaneously on the right and on the left side of the screen. An ellipsoid display format was used, in which all pieces of information (i.e., outcomes and probabilities) are present at equal distance from the initial fixation point (Figure 1). The ellipsoid format has been introduced in previous research, and it has been shown that choices are not systematically different from other more classic formats (see Glöckner and Betsch, 2008a; Glöckner and Herbold, 2011). The left (right) gamble was selected by pressing a key on the left (right) side of the keyboard (i.e., “y” and “m,” which are the lower left and right letters, respectively, on German keyboards). Decision tasks were shown in randomized order. The presentation order of the gambles (gamble is presented on the left or right side of the screen) and the order of outcomes within each gamble (i.e., low outcome first vs. high outcome first) were also chosen according to a fixed random assignment.

Eye movements and pupil dilations were recorded by using the eye gaze binocular system (LC Technologies) with remote binocular sampling rate of 120 Hz and an accuracy of about 0.45°. Gambles were presented on a monitor (Samsung Synchmaster 740B, refresh rate 60 Hz, reaction time 5 ms) with a native resolution of 1280 × 1024. Fixations were identified using a 30 pixel tolerance (i.e., added max-min deviation for *x*- and *y*-coordinates) and a minimum fixation time threshold of 50 ms. For analyses of mean fixation duration, first and last fixations in each decision trial were dropped since their length might be contaminated (see Krajbich et al., 2010; Glöckner and Herbold, 2011), although conclusions remained the same when including them in the analysis.

Non-overlapping AOI around the presented information on the screen were defined with the size of 100 × 100 pixels. These AOIs were used to determine which information was searched in a specific moment.

## Results

We first analyze dependent measures aggregated over the entire time course of decision making. In the second step, we look more closely at dynamics and systematic changes over time.

### Aggregated perspective

#### Choices

First, we were interested in the influence of EVdiff on choices; therefore we conducted logistic regressions predicting choice for Gamble A by the difference in EV between Gambles A and B (positive numbers indicating an advantage of A over B and vice versa for negative numbers). This and all following regressions use random effects models to account for the repeated measurement design. The estimated models include random intercepts and slopes for all relevant predictors (excluding control factors). Coefficients *c* are assumed to vary randomly and independently from each other on the level of participants around their population mean μ_C_ with a SD of σ_C_ following a normal distribution *N*[μ_C_, σ_C_] (Wooldridge, 2001)^5^. As a further control factor, we included trial number to account for order effects. We find coefficients for choosing Gamble A higher than 0, indicating that the probability for choosing this gamble increases with increasing EV difference in favor of the gamble (Table 2).

**Table 2:** **Logistic regression predicting choices for gamble A (*p*_choiceA_)**.

	*p*_choiceA_	*p*_choiceA_
	Study 1	Study 2
EVdiff_(Gamble A−Gamble B)_^a^	0.332*** (4.49)	0.801*** (12.63)
Constant	−0.124 (−0.98)	0.227* (2.13)

Observations	1026	1800

Random effects model with varying intercepts and slopes for EVdiff. Order effects have been included as control factors in the regression (not reported). Reported are raw coefficients (*z* statistics in parentheses); number of participants were *N* = 21 and 36 in Studies 1 and 2, respectively. *p < 0.05, ***p < 0.001. ^a^Values are in Euro difference.

#### The influence of mean EV and EV difference on information search and processing

We were first interested in the influence of our manipulations of EVmean and absolute EVdiff on information search and information integration. As dependent measures, we analyzed decision time, number of fixations, mean fixation duration (excluding first and last fixations), and pupil dilation. Time was measured from gamble onset until key-press, so was the number of fixations (i.e., count), and the mean fixation duration (i.e., averaged duration of single fixations over the time-frame). Pupil dilation was calculated as peak pupil dilation scores, that is, the maximum increase of pupil size from baseline (measured at blank screen and fixation cross before each decision) in the same time period. Pupil dilation is measured as radius in mm. Descriptive statistics for the core dependent measures are provided in Section “Summary Statistics” in Appendix.

All dependent measures were regressed on EVmean and absolute EVdiff using random effects models with random intercepts and random slopes for both predictors and trial as control factor (Table 3). In the second study, we find that decision time, number of fixations, mean fixation duration, and pupil dilation increase with EVmean. Regression coefficients provide quantitative estimates for the influence. An increase in mean EV of 1.00€, for example, increases decision time by 0.197 s, the number of fixations by 0.701, etc. Except for mean fixation duration, we find trends in the same directions in Study 1, which do not reach conventional levels of significance, however. It remains unclear here whether these non-significant results are caused by the lower power due to reduced sample size or other factors such as the more systematic gamble construction and the lower variation of EVmean in Study 1.

**Table 3:** **Regression models predicting decision time, number of fixations, fixation durations, and pupil dilation by EVmean and EVdiff (abs)**.

	Decision time^b^		Number of fixations		Mean fixation duration^c^		Pupil dilation	
	Study 1	Study 2	Study 1	Study 2	Study 1	Study 2	Study 1	Study 2
EVmean^a^	0.0196 (0.29)	0.197*** (5.64)	0.0184 (0.08)	0.701*** (5.62)	−0.0003 (−0.93)	0.0004*** (4.56)	0.0011 (0.88)	0.0016** (2.81)
EVdiff (abs)^a^	−0.715** (−2.95)	−0.93*** (−7.09)	−2.363** (−2.95)	−3.624*** (−7.26)	−0.0042*** (−4.21)	−0.0005 (−0.91)	0.0029 (0.56)	0.0015 (0.53)
Constant	8.891*** (−3.88)	8.871*** (14.92)	33.24*** (9.85)	33.31*** (−7.26)	0.199*** (26.36)	0.197*** (43.69)	0.107*** (5.20)	0.0743*** (7.40)

Observations	1005	1728	1004	1728	1002	1728	995	1682

Random effects model with varying intercepts and slopes for EVmean and EVdiff (abs). Order effects have been included as control factors in the regression (not reported). Reported are raw coefficients (*z* statistics in parentheses); number of participants were *N* = 21 and 36 in Studies 1 and 2, respectively. EVdiff (abs) denotes the absolute (i.e., none-negative) value of the difference between EVs of the gambles. **p < 0.01, ***p < 0.001. ^a^Values are in Euro (difference). ^b^Regression with log transformed decision times yield the same conclusions. ^c^The random effect model failed to calculate the SE and as a result did not provide a stable estimate of the coefficients. Therefore we report for mean fixation duration a fixed effect model with cluster corrected SE (results and interpretation stay the same for both analysis).

Furthermore, we find that decision time, and number of fixations increase with decreasing EVdiff (abs) between gambles in both studies. Mean fixation duration also increases with decreasing EVdiff (abs) in both studies but only in the first study the effect reaches conventional significance levels. An increase in EVdiff of 1 € would here result in an decrease in mean fixation duration of around 2%^6^.

To further investigate the effect of the EVmean manipulation on mean fixation durations, we categorized single fixations as short (<150 ms), medium (≥150 and <500 ms), and long (≥500 ms) according to their duration (Velichkovsky, 1999; Horstmann et al., 2009). In both studies (and in both high and low EVmean gambles) we find mainly medium and short fixations (Study 1: *M*_short_ = 42.09%, *M*_medium_ = 54.81%; Study 2: *M*_short_ = 41.91%, *M*_medium_ = 55.89%) and only a few long fixations that indicate more deliberate processing. Mixed effect regressions with proportion of short, medium, and long fixations as dependent variables and EV mean as predictor indicate that in both studies neither of the proportions is significantly influenced by EV mean, all *p*’s > 0.13.

#### Influence of probability and outcome on attention

To investigate driving factors for the distribution of attention, we regressed the amount of fixations toward each outcome on its probability, its value, and their interaction. As in the regression reported above, we corrected for the repeated measurement design by using a random effects model with random intercept and random slopes for all three predictors. Furthermore we corrected for display position (i.e., all four combinations of right/left × up/down) using three display position dummies as well as for learning effects over trials by including trial number. In both studies the amount of fixations spent on an outcome increases with both its probability and the value of the outcome (Table 4). The interaction of probability and value is significant in Study 2 and marginally significant in Study 1. Significant main effects (but no interactions) are also found in regressions using fixation time as dependent variable (not reported).

**Table 4:** **Regression models predicting fixations to outcomes by value and probability**.

	Number of fixations	
	Study 1	Study 2
Value^a^	0.0741*** (4.97)	0.0913*** (9.78)
Probability^b^	1.533*** (8.21)	1.707*** (8.30)
Value × probability	0.0685^+^ (2.03)	0.125*** (7.04)
Constant	5.147*** (10.63)	5.428*** (15.37)

Observations	3892	6793

Random effects model with varying intercepts and slopes for value and probability. Order and position effects have been included as control factors in the regression (not reported). Reported are raw coefficients (*z* statistics in parentheses); number of participants were *N* = 21 and 36 in Studies 1 and 2, respectively. ^+^*p* < 0.10, ***p < 0.001. ^a^Values are in Euro (centered). ^b^Probabilities vary between 0 and 1(centered).

**Figure 2:**
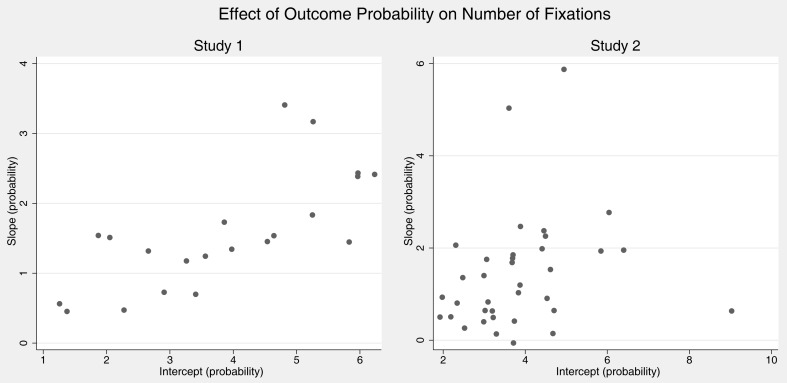
**Individual regression coefficients for predicting number of fixations by probability of outcomes (controlling for value)**. Graphs show intercepts plotted against slopes with positive slopes indicating that the number of fixation increases with probability.

**Figure 3:**
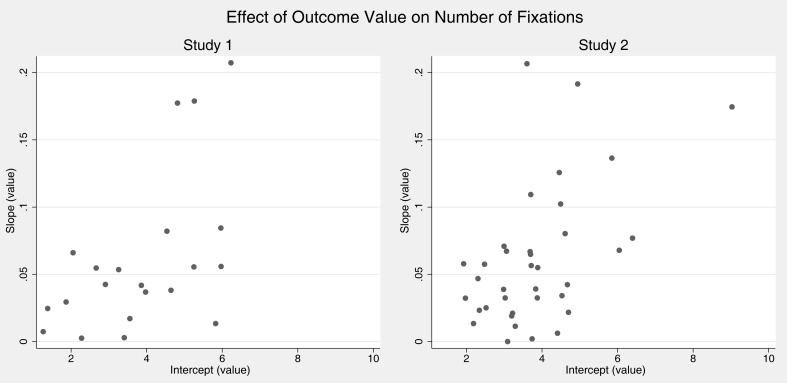
**Individual regression coefficients for predicting number of fixations by value of outcomes (controlling for probability)**. Graphs show intercepts plotted against slopes with positive slopes indicating that the number of fixation increases with value.

Some heuristics models assume interindividual differences in that persons use qualitatively different strategies to make risky choices (Payne et al., 1988, 1992; Gigerenzer and Todd, 1999). These should be reflected in heterogeneity of attention patterns between individuals. We conducted regressions per individual to investigate eventual heterogeneity in the effects of probabilities and outcomes on fixations. Figures 2 and 3 plot the resulting intercepts against the slopes from these regressions, each dot indicating one participant. We were thereby mainly interested in slopes. Interestingly, for both effects of probability and value on the number of fixations almost all participants showed behavior that was qualitatively in line with the overall regression results in that slopes for probabilities and values were positive. Hence, although slopes and intercepts differ between persons behavior seems to be relatively homogeneous.

### Dynamic perspective

Furthermore, we investigated dynamics over the course of decision making. Some models predict changes in attention. Heuristics, such as PH and LEX, predict changes of attention from more important to less important comparisons over time. Most strategies considered here except for PCS (and also the above mentioned attention drift-diffusion models), however, predict that attention should be about equally distributed over the two gambles that are compared. As introduced above, Glöckner and Herbold (2011) showed an attention bias toward the option chosen later on in risky choices and gaze-cascade effects, that is a tendency to increasingly look at options that are later on chosen, have been demonstrated repeatedly in decisions under certainty (e.g., Shimojo et al., 2003). We investigate whether we can replicate the attentional bias and aim to explore in more detail when the bias starts to occur in the decision process. We expect to find a gaze-cascade effect in risky choice, in that the attentional bias is particularly driven by late fixations.

#### Attentional bias

**Figure 4:**
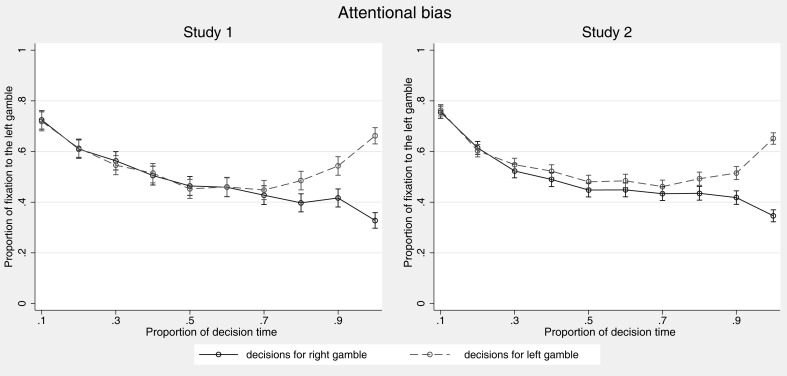
**Proportion of fixations to the left gamble (probabilities and outcomes) over the time course of the decision**. Decisions for right vs. left gamble are depicted by separate lines. Error bars indicate 95% confidence intervals.

To investigate the occurrence of an attentional bias over the course of decision making, the proportion of fixations to the left gamble is used as the dependent measure. Figure 4 plots the attention proportion against proportional time bins with each bin containing 10% of the absolute decision time per person and decision.

Descriptively, the attentional bias toward the favored gamble is replicated. In both studies, a strong separation can be seen in the last third of the decision process (i.e., starting in the seventh or eighth bin). In both studies, we also consistently see an initial attention bias to the left which is driven by reading direction.

To test the results statistically, we regressed the proportion of fixation to the left gamble on choice (left/right), proportion of time (i.e., time bins), and their interaction (Table 5). We ran a random effects regression with random coefficients for intercept and slopes for all three predictors and included dummies for decision tasks as control factors. The significant main effect for choice indicates an overall attention bias in the direction of the chosen option. That is, if the left gamble was chosen, it was fixated 5–6% more often as compared to decisions in which the right gamble was chosen. The significant main effect for proportion of time on fixations to the left gamble indicates a general shift of fixations from left to right by 22–23% over the time course of decision making, probably driven by initial left bias due to natural reading order. More importantly, the significant interaction between choice and proportion of time indicates a strong gaze-cascade effect and captures the fact that the attentional bias mainly appears in the last part of the decision process in which the gamble receives attention which is chosen later on (independent of whether it is presented left or right)^7^.

**Table 5:** **Regression model predicting proportion of fixations to the left gamble**.

	Proportion of fixations to the left gamble	
	Study 1	Study 2
Choice (0 = right 1 = left)	5.088*** (3.71)	5.950*** (8.10)
Proportion of time^a^	−22.97*** (−4.79)	−22.16*** (−6.11)
Choice × proportion of time	27.24*** (7.56)	22.70*** (9.08)
Constant	47.68*** (15.46)	53.32*** (25.06)

Observations	9564	16944

Random effects model with varying intercepts and slopes for choice, time, and the interaction. Task effects have been included as control factors in the regression (not reported). Reported are raw coefficients (*z* statistics in parentheses); number of participants were *N* = 21 and 36 in Studies 1 and 2, respectively. ***p < 0.001. The range of the variable is between 0 and 1 and we used bins of 0.10 for the analysis (both variables were centered for the analysis). Results stay essentially the same when excluding extreme values (i.e., *p*90, *p*10) or using fixation time as dependent measure.

#### Pupil dilation

**Figure 5:**
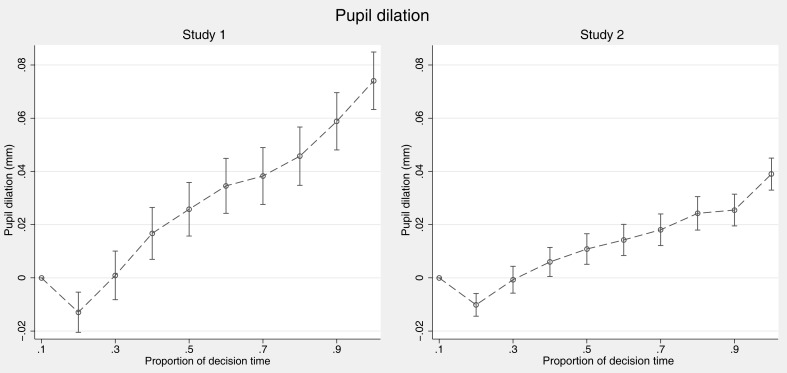
**Pupil dilation over time course of the decision**. Error bars indicate 95% confidence intervals (Note that SEs are estimated assuming independence within participants since deviation scores are normalized per person and trial).

As reported above, on the aggregate level, we found that pupil dilation increased with EVmean, that is, with the stakes involved in the decision (note, however, that the effect was significant in Study 2 only). We were now interested in the development of pupil dilation over time. The measure of dilation peaks used in the aggregated analysis above is inappropriate for such an analysis of small time-blocks (bins), since no single peaks per time-bin can be expected. We therefore calculated for each time-bin average deviation scores in pupil size from the first time-bin. Figure 5 shows that in both studies we observed an increase in pupil dilation over time. Additionally, there was an unexpected early drop in the second time-bin, which was observed in both studies and might eventually be caused by brightness changes due to stimulus onset.

To analyze pupil size development over time statistically, we regressed pupil dilation scores (i.e., pupil size minus pupil size in the first bin) on time, EVmean, absolute EVdiff, and their interactions with time (Table 6). We thereby again used a random effects model with intercept and slopes of all five predictors as random coefficients and controlling for order effects and the absolute time that passed within the trial. The analysis confirms that pupil dilation increases over the decision making process. The main effect for EVmean in Study 2 reproduces the effects from the overall analysis which used peak dilation scores (see Table 3, above).

**Table 6:** **Regression predicting changes in pupil dilation over the decision making process**.

	Pupil dilation	
	Study 1	Study 2
Proportion of time^a^	0.0434* (2.00)	0.0179^+^ (1.82)
EVmean^b^	0.000873 (0.80)	0.00105** (3.02)
EVdiff (abs)^b^	0.00369^+^ (1.93)	0.0027 (1.27)
Proportion of time × EVmean	0.00674 (1.39)	0.00703 (1.26)
Proportion of time × EVdiff (abs)	0.0140* (2.13)	0.00466(1.41)
Absolute time (in s)	0.00475*** (11.04)	0.0029*** (11.29)
Constant	0.0157 (11.04)	−0.00228 (−0.38)

Observations	9953	17427

Random effects model with varying intercepts and slopes for EVmean, EVdif(abs), time, and the interactions. Order effects have been included as control factors in the regression (not reported). Reported are raw coefficients (*z* statistics in parentheses); number of participants were *N* = 21 and 36 in Studies 1 and 2, respectively, ^+^*p* < 0.10, *p < 0.05, **p < 0.01, ***p < 0.001. ^a^The range of the variable is between 0 and 1 and we used bins of 0.10 for the analysis (and centered for the analysis). ^b^Values are in Euro (and centered for the analysis).

Both in the aggregated and the dynamic analysis, we do not find a negative effect of EVdiff (abs) on pupil dilation, which stands in conflict with the findings reported in the introduction of this paper and fails to support predictions derived from PCS. Note, however, that in Study 1, EVdiff (abs) is only manipulated for a small subset of the gambles (i.e., the Holt–Laury gambles), so that eventual effects might have been hard to detect.

#### Attention toward outcomes vs. probabilities

Changes in preferences for value and probability information during the decision can be informative for details of the decision process and were therefore analyzed as well. As mentioned above, some heuristics such as PH predict such shifts.

**Figure 6:**
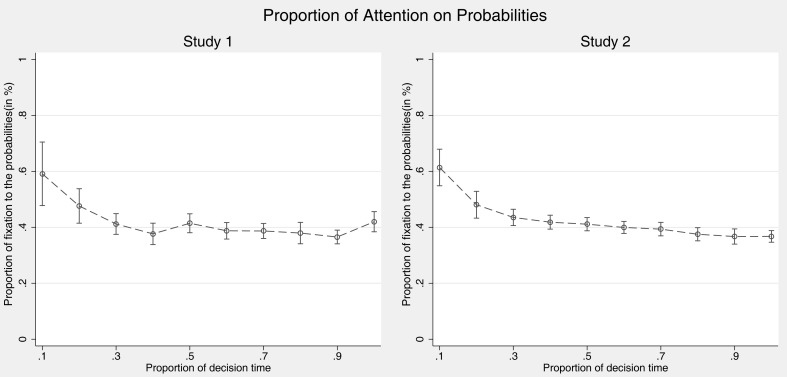
**Proportion of fixations to probabilities by time**. Error bars indicate 95% confidence intervals.

We analyzed the overall attention toward probabilities and outcomes of the gambles, as well as their development over time using the probability of fixations to probabilities as dependent measure (i.e., fixations to probability AOIs divided by fixations to all AOIs). As Figure 6 shows, participants show a preference for probabilities very early in the decision process and a preference for outcomes later on. This is in line with one possible interpretation of DFT, which puts forward that individuals first have to learn probabilities, which are used later on to guide fixations to outcomes (see text footnote 3, above).

To investigate these effects statistically, we regressed the proportion of fixations dedicated to probabilities on time-bin, again using a random effects model and controlling for task and order (Table 7). The constant indicates that there is a general preference for value information but the significant main effect points to an even stronger reversal from preferring probabilities to preferring value information over the course of decision making.

**Table 7:** **Regression model predicting proportion of fixation to probability information over time**.

	Proportion of fixations to probability information	
	Study 1	Study 2
Proportion of time (10%-steps)^a^	−14.69*** (−3.74)	−20.26*** (−7.61)
Constant	47.93*** (17.35)	49.62*** (23.90)

Observations	9428	16685

Random effects model with varying intercepts and slopes for time. Order effects have been included as control factors in the regression (not reported). Reported are raw coefficients (*z* statistics in parentheses); number of participants were *N* = 21 and 36 in Studies 1 and 2, respectively. ***p < 0.001. ^a^The range of the variable is between 0 and 1 and we used bins of 0.10 for the analysis.

#### Direction of information search

Finally, we investigated the direction of information search, which is one classic approach to investigate decision processes (e.g., Payne et al., 1988). As discussed above, WADD mainly predicts information search within-gambles, whereas heuristics mainly assume comparisons between gambles. We calculated the number of transitions within and between gambles as the number of times in which two subsequent fixations focused on different AOIs within the same gamble (within-gamble transition), as opposed to any AOI of the other gamble (between-gamble transition).

In line with previous findings (Franco-Watkins and Johnson, 2011; Glöckner and Herbold, 2011), we observe only a small proportion of transitions between gambles. Information search is mainly conducted within-gambles. We observe on average 5.12 (Study 1)/6.35 (Study 2) transitions between gambles and 14.44 (Study 1)/14.11 (Study 2) transitions within one of the gambles during each decision trial. We analyze changes in the proportion of transitions over the time course of decision making using a random effects model with randomly varying intercept and slope for the time trend. Findings for both studies indicate that there is no change in the proportion of fixations by time (both *z*’s < 1.16)^8^.

### Summary of results and theory predictions

A summary of the core results from the two reported eye-tracking studies with respect to the predictions of the theories that were considered in the current paper is provided in Table 8.

**Table 8:** **Summary of the results**.

Findings		Predicted by						
		DFT	PCS	PH	Minimax	Maximax	LEX	WADD
Decision time increases with	Higher EVmean	Yes	Yes	No	No	No	No	No
	Lower EVdiff	Yes	Yes	No	No	No	No	No
Average number of fixations increases with	Higher EVmean	Yes	Yes	No	No	No	No	No
	Lower EVdiff	Yes	Yes	No	No	No	No	No
Mean fixation duration increases with	Higher EVmean	Results are not conclusive						
	Lower EVdiff	Yes	Yes	No	No	No	No	No
Pupil dilation increases with	Higher EVmean	Yes	Yes	No	No	No	No	No
	Lower EVdiff	Results are not conclusive						
Attention increases with probability		Yes	Yes	No	No	No	Yes	No
Attention increases with value		No	Yes	No	No	Yes	No	No
Attention shift toward the chosen option		No	Yes	No	No	No	No	No
Mainly within-gambles information search		Unsp.	Unsp.	No	No	No	No	Yes
Pupil dilation increases over the time course of the decision		Yes	No	Unsp.	Unsp.	Unsp.	Unsp.	Unsp.

*Unsp. = unspecified*.

## Discussion

The current paper presents two studies which extend research on the cognitive mechanisms involved in risky choices with a special focus on dynamics, that is, changes in information search and processing over the time course of decision making. Eye-tracking is used to measure different indicators for information search and integration over time with the aim to test assumptions and predictions of existing process models for decision making to inspire further improvements and development within or outside the frameworks previously suggested.

In line with previous research (e.g., Glöckner and Betsch, 2008a; Ayal and Hochman, 2009; Glöckner and Herbold, 2011; Hilbig and Glöckner, 2011), we observed relatively short decision times, which speaks against the hypothesis that individuals deliberately integrate probabilities and outcomes according to a calculation of weighted sums. Note, that particularly in the second study we used randomly generated gambles that made calculations quite hard. Take for example the decision between Gamble A, which pays 9.3€ with 20% and 0.4€ otherwise vs. Gamble B which pays 3.8€ with 88% and 2.2€ otherwise. Even for mathematically skilled persons it seems hard to assume that they can deliberately calculate and compare the EVs of these Gambles within less than 10 s^9^. Nevertheless, participants’ choices are still significantly predicted by the difference in EV between gambles and both outcomes and probabilities influence information search for almost all participants, which indicates that persons take into account value and probabilities and seem to integrate them to some degree. Additionally, we find mainly short and medium fixations over the entire time course of decision making, which supports the notion of individuals at least partially utilizing automatic processes in risky choice. DFT and PCS models describe possible process implementation that approximate weighted integration without relying on the unreasonable assumption that individuals indeed calculate them in a serial manner.

Quick decision responses could, of course, also be explained by simple heuristics. Considering that Gigerenzer and Gaissmaier (2011) start their definition of heuristics as follows: “A heuristic is a strategy that ignores part of the information…,” the high average proportion of inspected information (i.e., 93%) speaks against this explanation. Also the interpretation that persons scan all pieces of information but later on ignore parts of it in their decision process seems to conflicts with the data since probabilities and values of outcomes influence attention for almost all participants. Furthermore, individuals show mainly within-gamble information search and are sensitive to manipulations of EV which also speaks against the application of simple heuristics.

We manipulated mean EV and EV difference between gambles of the decision problems to investigate whether individuals’ reactions to these manipulations can be better explained by one of the models considered. Indicators for information search and processing are influenced by both manipulations. Deciding about gambles with somewhat higher stakes (i.e., higher mean EV) results in longer decision times, a more comprehensive information search (amount of fixations), deeper processing (mean length of a single fixation), and signs of increased arousal and/or cognitive load (greater pupil dilation). However, not only the stakes of a gamble influence the processing, but also the overall similarity of the two gambles in the choice pair, as indicated by the absolute EV difference. Deciding between two gambles that are very close to each other with respect to EV was associated with an increase in decision times and number of fixations, as well as a larger average fixation duration. Summing up the evidence, we observe that individuals make choices in less then 10 s and thereby take into account almost all pieces of information. Choice behavior is thereby highly sensitive to manipulations in EVmean and EVdiff, which arguably requires individuals quickly to develop rough impressions of the EVs of the gambles considered.

Since individuals seem to integrate many available pieces of information in a fast automatic response, these results provide evidence in favor of automatic integration models such as PCS and DFT. Results do not fit with the assumptions of simple heuristics ignoring parts of the information presented, or the much slower serial integration of information proposed by WADD.

Since most process models provide predictions about the distribution of attention, we used the amount of fixations on each outcome as a dependent measure. We were able to show that the look-up rate of an outcome depends on the value and the probability of an outcome and that most individuals show these effects. This result is challenging not only for heuristics, which often predict that attention is mainly driven by the value of an outcome (i.e., minimax and maximax), but also for models like DFT which assume that the attention to an outcome is only driven by its probability. Only PCS among the models considered here, which predicts holistic integration of all presented information, could account for the influence of both value and probability of an outcome on the attention devoted to it.

The results from both of our studies as well as evidence from previous research (Glöckner and Betsch, 2008a; Johnson et al., 2008; Franco-Watkins and Johnson, 2011; Glöckner and Herbold, 2011), show that within-gamble comparisons were much more frequent than between-gamble comparisons in these two-outcome risky choice tasks. This result supports WADD, but speaks against simple heuristics, which assume mainly attribute-wise comparisons between the gambles. DFT and PCS do not predict a specific direction of information search, and we suggest extending and specifying them by adding testable models of information search to increase their empirical content (Glöckner and Betsch, 2011).

### Dynamic perspective

A more in-depth analysis of attention processes across the time course of decision making is unique to this study. To pin-point dynamics in the process, we analyzed changes in the distribution of attention between outcomes and probabilities, changes in pupil dilation, as well as the attention biases toward one of the options as the decision process unfolded.

Individuals preferred looking at probability information in the very beginning of a decision. During the middle and later parts of the decision, however, individuals focused more strongly on outcome information. This shift in attention toward outcome information is in line with the assumption of DFT that probability information informs later on sampling of outcomes. It is hard to explain with most simple heuristics. PCS does not make clear predictions concerning this shift.

We also observed an attention bias toward the preferred option occurring in the later part of decision making showing the gaze-cascade effect for risky choices. This could be due to fixations toward the later chosen gamble within the decision making process or fixations in order to confirm the choice. Furthermore, pupil dilations increased over time in both studies, which can be interpreted as an accumulation of arousal over the information search process, which could be explained by DFT.

### Models for risky choices

The evidence presented in this paper advances our understanding of the time course of risky choice as well as the underlying processes. Results generally seem to support automatic integration models. Nevertheless, neither DFT nor PCS can in their current specifications fully account for all findings. In particular, with regard to DFT, the assumption of a stochastic sampling and evidence accumulation process directed by probabilities of outcomes can only be partially supported. The results at hand show that the probability of an outcome occurring is an important factor for the distribution of attention as predicted by DFT. However, it is not the only factor influencing information sampling. The value of an outcome influences the allocation of attention as well. Some implementations of DFT also assume that the allocation of attention between gambles should be constant over the entire decision process, which is not in line with our finding of an emerging bias toward the actually chosen option over time (i.e., the gaze-cascade effect). Other implementations of evidence accumulation models such as the attention drift-diffusion model (e.g., Krajbich et al., 2012), however, could account for such effects, at least when they concern last fixations only.

Concerning PCS the results are somewhat equivocal as well. The results concerning the attention bias and the gaze-cascade effect in risky choice strongly support the idea of systematic information distortions, particularly the accentuation of initial advantages of one option over time (Thagard, 1989; Holyoak and Simon, 1999; Simon et al., 2004; Glöckner and Herbold, 2011). Also the findings concerning the effects of EVmean on information search and arousal are in line with the models predictions and replicate and extend previous findings. However, it has to be qualified that the by PCS predicted and previously observed (Glöckner et al., 2012) effect of EVdiff on arousal was not found in the current studies. Also the observed increase in arousal over the time course of decision making is hard to explain with the PCS model. Furthermore, it has to be criticized that the PCS framework is currently not sufficiently specified to predict all information search parameters. Therefore, it seems necessary to extend the model by modeling information search more explicitly. The current findings, which provide a closer view of the choice process, can inform such model developments.

### Caveats and further research

As already addressed in the introduction, one concern with regards to the method used in our experiment and the interpretations derived from our results could be that data from eye-tracking potentially provides only a vague proxy for the information search and processing, because it might neglect internal processes of attention and information retrieval. This would, for example, be the case if individuals look-up information only once and retrieve it from memory at a later stage of the decision making process. However, two arguments can be made against this objection: First, the current design (as opposed, for example, to mouselab) enabled effortless visual retrieval of information, making retrieval from memory unnecessary. Second, participants did actually constantly and systematically sample visual information across the entire decision process. These considerations allow for more faith in the methods, findings, and conclusions of the current study.

We could not replicate the previously observed effect of EV differences on arousal (Glöckner et al., 2012). For deeper understanding, we therefore strongly encourage further studies of the link between the decision task difficulty and observed arousal or cognitive load. Investigating the connection with additional measures, like skin conductance, could help to clarify the role of arousal and the driving factors.

Besides investigations of a methodological nature, a next step in analyzing information search and arousal should be replications of these findings in different decision making contexts by, on the one hand, changing the structure of the gamble tasks into more than two-outcome gambles and, on the other hand, using less abstract risky choices in order to test whether the result also holds for affect richer decisions and can therefore be generalized to risky decisions in the “real world”(see i.e., Goldstein and Weber, 1995, for a discussion of this caveat).

## Conclusion

Given our results and evidence from previous studies (see Glöckner and Herbold, 2011), simple non-compensatory models such as the PH, LEX, minimax, or maximax heuristic do not seem to be appropriate to predict search behavior and processes involved in risky decisions in general. The same holds for the serial implementations of EU models in the form of WADD. Instead, the present results suggest that risky decision making seems to rely mainly on automatic-intuitive processes and can be partially described by models such as DFT and PCS. Nevertheless, also these models cannot account for all our findings in that they are underspecified in some respects and make no predictions or even make predictions which are clearly not in line with the findings. Since none of our findings, however, directly challenges core properties of these models, and also due to a lack of better alternatives, we think that both kinds of models are promising starting points for further theory developments concerning process models of risky choice.

## Conflict of Interest Statement

The authors declare that the research was conducted in the absence of any commercial or financial relationships that could be construed as a potential conflict of interest.

## Supplementary Material

The Supplementary Material for this article can be found online at http://www.frontiersin.org/Cognitive_Science/10.3389/fpsyg.2012.00335/abstract

## Footnotes

^1^For important limitations of CPT particularly in predicting choices between gambles with more than two outcomes see Birnbaum (2006, 2008a,b).

^2^The threshold is assumed to model the importance of a decision (e.g., Johnson and Busemeyer, 2005, p. 847) and arguably subjective importance increases with the stakes that are in place in a decision. Johnson and Busemeyer (2005, p. 843) furthermore state that: “Each moment in the DFT choice process is akin to mentally sampling one of these numbers [i.e., monetary outcomes of gambles], producing an affective reaction to the imagined result” (explanation in brackets are added). Everything else being equal (e.g., drift rate), scaling up stakes of a decision should therefore increase the amount of affective reactions that has to be sampled and accumulated. Besides this eventual effect of increased accumulation, the increased size of outcomes *per se* should lead to increased affective reaction for each sampled outcome as well. Given the well established link between the (absolute) strength of affective responses and pupil dilation (e.g., Janisse, 1974), both effects could cause larger pupil dilations. It, however, has to be acknowledged that the researchers that suggested DFT have (to the best of our knowledge) not explicitly derived these predictions concerning arousal from their theory.

^3^According to DFT, attention weights (often equated with probabilities) are assumed to determine the stochastic sampling process of outcomes. Therefore, “the outcome probabilities dictate where attention shifts, but only the outcome values are used in determining the momentary evaluation.” (Johnson and Busemeyer, 2005, p. 843). Note, however, that DFT in its original formulation is mainly concerned with mentally sampling outcomes. Explicit predictions concerning overt attention are only found in some implementation of simplified evidence accumulation models (e.g., Raab and Johnson, 2007), while other authors have argued that the process is not directly observable (e.g., Diederich, 2003).

^4^These gambles were also used in further eye-tracking studies by Glöckner and Herbold (2011) and Franco-Watkins and Johnson (2011) and comparative model fitting (together with additional gambles) in Glöckner and Pachur (2012).

^5^Alternative analyses using fixed effect correction and cluster corrected SE lead to the same conclusions except where explicitly stated otherwise.

^6^We also run all analyses reported in Table 4 including additional interaction effects of EVmean and EVdiff (abs). As expected, main effects did not change but interactions turned out to be significant and positive for mean fixation duration in Study 1 and for decision time and number of fixations in Study 2.

^7^For food choices, Krajbich et al. (2012) additionally report the effect that participants tend to choose the unlooked-at item if the last fixation was to a much disliked item. To investigate whether this effect generalizes to gambles, we rerun the regressions reported in Table 5 additionally including a three-way interaction of EVdiff, choice, and time (also controlling for the respective main effect for EVdiff and the remaining two-way interaction; all variables centered). The results indicate that there is a positive interaction effect in study 2 but not study 1, whereby main effects and other interaction effects are not significantly changed. This lack of support, however, might be due to the fact that there were no losses involved in our material.

^8^In this analysis, the application of a fixed effects model leads to different conclusions in that within gamble transitions increase over time in Study 2 (*b* = 2.8, *z* = 3.01, *p* < 0.001). Due to the inconsistency in analyses this result has to be interpreted cautiously. Note also that the finding conflicts with an opposite results by Glöckner and Herbold (2011).

^9^Note, that our manipulation of EVmean was partially confounded with mathematical complexity when assuming deliberate serial calculation of EVs. Specifically, gamble comparisons with high as compared to low mean EV also involve higher numbers that could make calculation harder and therefore increase decision time, number of fixations and arousal. Although we cannot completely rule out this possibility, we argue that given our findings concerning choices, decision time and process measures deliberate stepwise calculations of EV are not very likely in the first place.

## Appendix

### Summary statistics

Table A1 provides the descriptive statistics for the core dependent measures.

**Table A1:** **Summary statistic for the main dependent variables (SD)**.

	Study 1	Study 2
Pupil size (radius in mm)	2.49 (0.43)	2.26 (0.30)
Pupil dilation (radius in mm)	0.122 (0.133)	0.095 (0.11)
Decision time (in s)	8.98 (6.27)	9.29 (5.99)
Proportion of inspected information (in %)^a^	93.92 (13.57)	96.01 (9.42)
Mean fixation duration (in s)	0.196 (0.040)	0.194 (0.033)
Number of fixations per decision	33.48 (21.01)	34.99 (21.26)
Number of transitions between information pieces	19.35 (11.56)	20.33 (11.99)
Transitions within-gambles (in %)^b^	74.15 (13.81)	69.48 (13.75)
Proportion of long fixations (in %)^c^	3.08 (1.73)	2.20 (1.4)

*^a^Proportion of AOIs fixated per trial, eight pieces of information are presented each trial*.

*^b^Reference class are all direct transitions between AOIs, re-fixations within the same AOI are dropped*.

*^c^Long fixations are fixation >500 ms and the reference class are all identified fixations*.

### Gambles study 1

Table A2 shows the 50 decision tasks used in study 1. Each Choice consists of two gambles A and B with two possible outcomes (and their probabilities). In the presentation, the positions of gambles and outcomes were varied according to a fixed random design.

**Table A2:** **Decision tasks used in study 1**.

Decision	Gamble A	Gamble B	EV_A_	EV_B_	EVmean	EVdiff (abs)
1	0€ (0.60)/20€ (0.40)	10€ (0.40)/6.67€ (0.60)	8	8	8	0
2	4€ (0.50)/3€ (0.50)	0€ (0.30)/5€ (0.70)	3.5	3.5	3.5	0
3	1€ (0.45)/4€ (0.55)	4.4€ (0.60)/0€ (0.40)	2.65	2.64	2.645	0.01
4	10€ (0.7)/4€ (0.3)	10.25€ (0.80)/0€ (0.20)	8.2	8.2	8.2	0
5	10.5€ (0.50)/0.5€ (0.50)	11€ (0.75)/0€ (0.25)	8.25	8.25	8.25	0
6	0€ (0.30)/12€ (0.70)	10€ (0.60)/6€ (0.40)	8.4	8.4	8.4	0
7	7.5€ (0.50)/0€ (0.50)	2€ (0.65)/7€ (0.35)	3.75	3.75	3.75	0
8	3.3€ (0.40)/2.25€ (0.60)	4€ (0.67)/0€ (0.33)	2.67	2.68	2.675	0.01
9	0€ (0.40)/16€ (0.60)	22.5€ (0.40)/1€ (0.60)	9.6	9.6	9.6	0
10	4.5€ (0.70)/0€ (0.30)	0.3€ (0.50)/6€ (0.50)	3.15	3.15	3.15	0
11	5€ (0.60)/0€ (0.40)	0.4€ (0.55)/6.2€ (0.45)	3	3	3	0
12	11.5€ (0.70)/0.6€ (0.30)	10.3€ (0.80)/0€ (0.20)	8.23	8.24	8.235	0.01
13	0€ (0.25)/11.35€ (0.75)	16.25€ (0.50)/0.75€(0.50)	8.51	8.5	8.505	0.01
14	0.4€ (0.60)/7.3€ (0.40)	0€ (0.30)/4.5€ (0.70)	3.16	3.15	3.155	0.01
15	0€ (0.50)/4€ (0.50)	0.39€ (0.65)/5€ (0.35)	2	2	2	0
16	11.27€ (0.67)/0€ (0.33)	0.6€ (0.60)/18€ (0.40)	7.55	7.56	7.555	0.01
17	2€ (0.50)/3.4€ (0.50)	2.5€ (0.60)/3€ (0.40)	2.7	2.7	2.7	0
18	2€ (0.60)/5.25€ (0.40)	3€ (0.70)/4€ (0.30)	3.3	3.3	3.3	0
19	6.6€ (0.55)/11€ (0.45)	8.8€ (0.60)/8.25€ (0.40)	8.58	8.58	8.58	0
20	10€ (0.75)/6€ (0.25)	10€ (0.50)/8€ (0.50)	9	9	9	0
21	9€ (0.50)/7.5€ (0.50)	5€ (0.35)/10€ (0.65)	8.25	8.25	8.25	0
22	4.4€ (0.65)/15.4€ (0.35)	5.5€ (0.50)/11 € (0.50)	8.25	8.25	8.25	0
23	5.25€ (0.40)/2€ (0.60)	4€ (0.30)/3€ (0.70)	3.3	3.3	3.3	0
24	3.45€ (0.50)/1€ (0.50)	0.5€ (0.60)/4.8 € (0.40)	2.22	2.22	2.22	0
25	49.5€ (0.10)/5.5€ (0.90)	10€ (0.99)/0€ (0.01)	9.9	9.9	9.9	0
26	0€ (0.02)/9.44 € (0.98)	4.7€ (0.85)/35 € (0.15)	9.25	9.25	9.25	0
27	1.35€ (0.90)/12.5€ (0.10)	0€ (0.01)/2.5€ (0.99)	2.46	2.47	2.47	0.01
28	0€ (0.02)/3€ (0.98)	13.1€ (0.1)/1.8€ (0.9)	2.94	2.93	2.94	0.01
29	34€ (0.15)/3.5€ (0.85)	8.3€ (0.98)/0.1€ (0.02)	8.07	8.14	8.11	0.07
30	2.25€ (0.98)/0.1€ (0.02)	9€ (0.15)/1€ (0.85)	2.21	2.2	2.20	0.01
31	2.5€ (0.99)/0.3€ (0.01)	12.2€ (0.10)/1.4€ (0.90)	2.48	2.48	2.48	0
32	9€ (0.98)/0€ (0.02)	34.3€ (0.10)/6€ (0.90)	8.82	8.83	8.825	0.01
33	2.9€ (0.98)/3.2€ (0.02)	1.3€ (0.85)/12€ (0.15)	2.91	2.91	2.91	0
34	3.8.€ (0.85)/34 € (0.15)	8.3€ (0.98)/10 € (0.02)	8.33	8.33	8.33	0
35	1.44€ (0.85)/12€ (0.15)	3€ (0.98)/4€ (0.02)	3.02	3.02	3.02	0
36	4.58€ (0.80)/22€ (0.20)	15€ (0.01)/8€ (0.99)	8.07	8.07	8.07	0
37	17.2€ (0.15)/0.5€ (0.85)	3.2€ (0.01)/3€ (0.99)	3	3	3	0
38	31.2€ (0.20)/2.8€ (0.80)	8.5€ (0.99)/7.5€ (0.01)	8.48	8.49	8.485	0.1
39	0.9€ (0.80)/33.9€ (0.20)	8€ (0.01)/7.5€ (0.99)	7.5	7.5	7.5	0
40	22.5€ (0.15)/0.5€ (0.85)	2.15€ (0.98)/2.3€ (0.02)	3.8	2.153	2.98	1.65
41	3.2€ (0.90)/4€ (0.10)	7.7€ (0.10)/0.2€ (0.90)	3.28	0.95	2.12	2.33
42	3.2€ (0.80)/4€ (0.20)	7.7€ (0.20)/0.2€ (0.80)	3.36	1.7	2.53	1.66
43	3.2€ (0.70)/4€ (0.30)	7.7€ (0.30)/0.2€ (0.70)	3.44	2.45	2.95	0.99
44	3.2€ (0.60)/4€ (0.40)	7.7€ (0.40)/0.2€ (0.60)	3.52	3.2	3.36	0.32
45	3.2€ (0.50)/4€ (0.50)	7.7€ (0.50)/0.2€ (0.50)	3.6	3.95	3.78	0.35
46	3.2€ (0.40)/4€ (0.60)	7.7€ (0.60)/0.2€ (0.40)	3.68	4.7	4.19	1.02
47	3.2€ (0.30)/4€ (0.70)	7.7€ (0.70)/0.2€ (0.30)	3.76	5.45	4.61	1.69
48	3.2€ (0.20)/4€ (0.80)	7.7€ (0.80)/0.2€ (0.20)	3.84	6.2	5.02	2.36
49	3.2€ (0.10)/4€ (0.90)	7.7€ (0.90)/0.2€ (0.10)	3.92	6.95	5.44	3.03
50	3.2€ (0.00)/4€ (0.1)	7.7€ (1)/0.2€ (0.00)	4	7.7	5.85	3.70

### Gambles study 2

Table A3 shows the 50 decision tasks used in study 2. Each Choice consists of two gambles A and B with two possible outcomes (and their probabilities). In the presentation, the positions of gambles and outcomes were varied according to a fixed random design.

**Table A3:** **Decision tasks used in study 2**.

Decision	Gamble A	Gamble B	EV_A_	EV_B_	EVmean	EVdiff (abs)
1	2.4€ (0.75)/0.8 € (0.25)	3.4€ (0.49)/0.8 € (0.51)	2	2.07	2.04	0.07
2	2.2€ (0.82)/2.8€ (0.18)	2€ (0.16)/2.3€ (0.84)	2.31	2.25	3.44	0.06
3	9.5€ (0.2)/0.6€ (0.8)	2.6€ (0.88)/1€ (0.12)	2.38	2.41	2.40	0.03
4	1€ (0.72)/7.8€ (0.28)	2.3€ (0.77)/4.6€ (0.23)	2.90	2.83	2.87	0.07
5	1.2€ (0.76)/5.3€ (0.24)	0.9€ (0.2)/2.4 € (0.8)	2.18	2.10	2.14	0.08
6	7€ (0.23)/1.5€ (0.77)	1.9€ (0.85)/8.1€ (0.15)	2.77	2.83	2.80	0.06
7	1.8€ (0.03)/2.4€ (0.97)	2.5€ (0.69)/2.1€ (0.31)	2.38	2.38	2.38	0
8	6.8€ (0.18)/1€ (0.82)	3.9€ (0.14)/1.8€ (0.86)	2.04	2.09	2.07	0.05
9	2.7€ (0.66)/2.9€ (0.34)	1.5€ (0.73)/6.3€ (0.27)	2.77	2.80	2.79	0.03
10	4€ (0.1)/1.9€ (0.9)	6.2€ (0.16)/1.3€ (0.84)	2.11	2.08	2.10	0.03
11	14.4€ (0.75)/4.8€ (0.25)	20.4€ (0.49)/4.8€ (0.51)	12	12.44	12.22	0.44
12	13.2€ (0.82)/16.8€ (0.18)	12€ (0.16)/13.8€ (0.84)	13.85	13.51	13.68	0.34
13	57€ (0.2)/3.6€ (0.8)	15.6€ (0.88)/6€ (0.12)	14.28	14.45	14.37	0.17
14	6€ (0.72)/46.8€ (0.28)	13.8€ (0.77)/27.6€ (0.23)	17.42	16.97	17.20	0.45
15	7.2€ (0.76)/31.8€ (0.24)	5.4€ (0.2)/14.4€ (0.8)	13.10	12.60	12.85	0.50
16	42€ (0.23)/9€ (0.77)	11.4€ (0.85)/48.6€ (0.15)	16.59	16.98	16.79	0.39
17	10.8€ (0.03)/14.4€ (0.97)	15€ (0.69)/12.6€ (0.31)	14.29	14.26	14.28	0.04
18	40.8€ (0.18)/6€ (0.82)	23.4€ (0.14)/10.8 € (0.86)	12.26	12.56	12.41	0.30
19	16.2€ (0.66)/17.4€ (0.34)	9€ (0.73)/37.8 € (0.27)	16.61	16.78	16.69	0.17
20	24€ (0.1)/11.4€ (0.9)	37.2€ (0.16)/7.8€ (0.84)	12.66	12.50	12.58	0.16
21	2.2€ (0.75)/0.6€ (0.25)	4.6€ (0.49)/2€ (0.51)	1.80	3.27	2.54	1.47
22	2€ (0.82)/2.6€ (0.18)	3.2€ (0.16)/3.5€ (0.84)	2.11	3.45	2.78	1.34
23	9.3€ (0.2)/0.4€ (0.8)	3.8€ (0.88)/2.2€ (0.12)	2.18	3.61	2.90	1.43
24	0.8€ (0.72)/7.6€ (0.28)	3.5€ (0.77)/5.8€ (0.23)	2.70	4.03	3.40	1.33
25	1€ (0.76)/5.1€ (0.24)	2.1€ (0.2)/3.6€ (0.8)	1.98	3.30	2.64	1.32
26	6.8€ (0.23)/1.3€ (0.77)	3.1€ (0.85)/9.3€ (0.15)	2.57	4.03	3.30	1.46
27	1.6€ (0.03)/2.2€ (0.97)	3.7€ (0.69)/3.3€ (0.31)	2.18	3.58	2.88	1.40
28	6.6€ (0.18)/0.8€ (0.82)	5.1€ (0.14)/3€ (0.86)	1.84	3.29	2.57	1.45
29	2.5€ (0.66)/2.7€ (0.34)	2.7€ (0.73)/7.5€ (0.27)	2.57	4	3.28	1.43
30	3.8€ (0.1)/1.7€ (0.9)	7.4€ (0.16)/2.5€ (0.84)	1.91	3.28	2.60	1.37
31	14.2€ (0.75)/4.6€ (0.25)	21.6€ (0.49)/6€ (0.51)	11.80	13.64	12.72	1.84
32	13€ (0.82)/16.6€ (0.18)	13.2€ (0.16)/15€ (0.84)	13.65	14.71	14.18	1.06
33	56.8€ (0.2)/3.4€ (0.8)	16.8€ (0.88)/7.2€ (0.12)	14.08	15.65	14.87	1.57
34	5.8€ (0.72)/46.6€ (0.28)	15€ (0.77)/28.8 € (0.23)	17.22	18.17	17.70	0.95
35	7€ (0.76)/31.6 € (0.24)	6.6€ (0.2)/15.6€ (0.8)	12.90	13.80	13.35	0.90
36	41.8€ (0.23)/8.8€ (0.77)	12.6€ (0.85)/49.8 € (0.15)	16.39	18.18	17.30	1.79
37	10.8€ (0.03)/14.2€ (0.97)	16.2€ (0.69)/13.8 € (0.31)	14.09	15.46	14.77	1.36
38	40.6€ (0.18)/5.8€ (0.82)	24.6€ (0.14)/12€ (0.86)	12.06	13.76	12.91	1.7
39	16€ (0.66)/17.2€ (0.34)	10.2€ (0.73)/39€ (0.27)	16.41	17.98	17.20	1.57
40	23.8€ (0.1)/11.2€ (0.9)	38.4€ (0.16)/9€ (0.84)	12.46	13.70	13.08	1.24
41	3.2€ (0.90)/4€ (0.10)	7.7€ (0.10)/0.2€ (0.90)	3.28	0.95	2.12	2.33
42	3.2€ (0.80)/4€ (0.20)	7.7€ (0.20)/0.2€ (0.80)	3.36	1.70	2.53	1.66
43	3.2€ (0.70)/4€ (0.30)	7.7€ (0.30)/0.2€ (0.70)	3.44	2.45	3	0.99
44	3.2€ (0.60)/4€ (0.40)	7.7€ (0.40)/0.2€ (0.60)	3.52	3.20	3.36	0.32
45	3.2€ (0.50)/4€ (0.50)	7.7€ (0.50)/0.2€ (0.50)	3.60	3.95	3.78	0.35
46	3.2€ (0.40)/4€ (0.60)	7.7€ (0.60)/0.2€ (0.40)	3.68	4.70	4.19	1.02
47	3.2€ (0.30)/4€ (0.70)	7.7€ (0.70)/0.2€ (0.30)	3.76	5.45	4.61	1.69
48	3.2€ (0.20)/4€ (0.80)	7.7€ (0.80)/0.2€ (0.20)	3.84	6.20	5.02	2.36
49	3.2€ (0.10)/4€ (0.90)	7.7€ (0.90)/0.2€ (0.10)	3.92	6.95	5.44	3.03
50	3.2€ (0.00)/4€ (0.1)	7.7€ (1)/0.2€ (0.00)	4	7.70	5.85	3.70
